# Supplementary material for: The effect of ULV-based mosquito control on target and non-target organisms in Hungary: An experimental field study
Source: PLoS Negl Trop Dis. 2026 Oct 5;20(10):e0014140. doi: 10.1371/journal.pntd.0014140 (PMC13657401; doi:10.1371/journal.pntd.0014140)
Supplement: Supplementary file 1 [file pntd.0014140.s001.docx]

**S1 Table.** Results of the mixed models testing for the effect of treatment on mosquito abundance (number of individuals) when accounting for the hierarchical structure of data and year and date effects.

| Treatment group |  | |  |  |  |
| --- | --- | --- | --- | --- | --- |
| Fixed effects | Estimate | S.E. | | t-value |  |
| Intercept | 2.052 | 0.876 | | 2.343 |  |
| status (before or after the treatment) | 0.367 | 0.083 | | 4.447 |  |
| year | -0.123 | 0.257 | | -0.481 |  |
| date | -0.003 | 0.004 | | -0.773 |  |
|  |  |  | |  |  |
| Random effects |  |  | |  |  |
| *Variance parameters* |  |  | |  |  |
| site |  |  | |  | 0.066 |
| locality |  |  | |  | 0.022 |
| residual |  |  | |  | 0.075 |
| *Dimensions* |  |  | |  |  |
| # of sites. |  |  | |  | 22 |
| # of localities |  |  | |  | 5 |

Significance of fixed effects based on likelihood ratio tests: status, *P* < 0.001; year, *P* = 0.631; date, *P* = 0.439

| Control group |  | |  |  |  |
| --- | --- | --- | --- | --- | --- |
| Fixed effects | Estimate | S.E. | | t-value |  |
| Intercept | 0.388 | 13.129 | | 0.030 |  |
| status (before or after the treatment) | 0.142 | 0.125 | | 1.131 |  |
| year | 0.348 | 2.326 | | 0.150 |  |
| date | 0.003 | 0.058 | | 0.049 |  |
|  |  |  | |  |  |
| Random effects |  |  | |  |  |
| *Variance parameters* |  |  | |  |  |
| site |  |  | |  | < 0.001 |
| locality |  |  | |  | 0.315 |
| residual |  |  | |  | 0.063 |
| *Dimensions* |  |  | |  |  |
| # of sites. |  |  | |  | 8 |
| # of localities |  |  | |  | 5 |

Significance of fixed effects based on likelihood ratio tests: status, *P* = 0.258; year, *P* = 0.881; date, *P* = 0.961
