# Supplementary material for: The effect of ULV-based mosquito control on target and non-target organisms in Hungary: An experimental field study
Source: PLoS Negl Trop Dis. 2026 Oct 5;20(10):e0014140. doi: 10.1371/journal.pntd.0014140 (PMC13657401; doi:10.1371/journal.pntd.0014140)
Supplement: Supplementary file 2 [file pntd.0014140.s002.docx]

**S2 Table.** Results of the mixed models testing for the effect of treatment on the abundance of pollinators when accounting for the hierarchical structure of data and year and date effects.

| Treatment group |  | |  |  |  |
| --- | --- | --- | --- | --- | --- |
| Fixed effects | Estimate | S.E. | | t-value |  |
| Intercept | 0.685 | 1.096 | | 0.625 |  |
| status (before or after the treatment) | 0.176 | 0.055 | | 3.223 |  |
| year | 0.371 | 0.275 | | 1.351 |  |
| date | < 0.001 | 0.005 | | 0.082 |  |
|  |  |  | |  |  |
| Random effects |  |  | |  |  |
| *Variance parameters* |  |  | |  |  |
| site |  |  | |  | 0.189 |
| locality |  |  | |  | 0 |
| residual |  |  | |  | 0.031 |
| *Dimensions* |  |  | |  |  |
| # of sites. |  |  | |  | 21 |
| # of localities |  |  | |  | 5 |

Significance of fixed effects based on likelihood ratio tests: status, *P* = 0.001; year, *P* = 0.176; date, *P* = 0.934

| Control group |  | |  |  |  |
| --- | --- | --- | --- | --- | --- |
| Fixed effects | Estimate | S.E. | | t-value |  |
| Intercept | 13.265 | 11.044 | | 1.201 |  |
| status (before or after the treatment) | 0.198 | 0.247 | | 0.805 |  |
| year | -2.085 | 1.994 | | -1.046 |  |
| date | -0.053 | 0.049 | | -1.089 |  |
|  |  |  | |  |  |
| Random effects |  |  | |  |  |
| *Variance parameters* |  |  | |  |  |
| site |  |  | |  | 0.376 |
| locality |  |  | |  | < 0.001 |
| residual |  |  | |  | 0.182 |
| *Dimensions* |  |  | |  |  |
| # of sites. |  |  | |  | 6 |
| # of localities |  |  | |  | 4 |

Significance of fixed effects based on likelihood ratio tests: status, *P* = 0.421; year, *P* = 0.296; date, *P* = 0.276
