# Supplementary material for: The effect of ULV-based mosquito control on target and non-target organisms in Hungary: An experimental field study
Source: PLoS Negl Trop Dis. 2026 Oct 5;20(10):e0014140. doi: 10.1371/journal.pntd.0014140 (PMC13657401; doi:10.1371/journal.pntd.0014140)
Supplement: Supplementary file 3 [file pntd.0014140.s003.docx]

**S3 Table.** Results of the mixed models testing for the effect of treatment on the abundance of all insects that were captured in the malaise traps when accounting for the hierarchical structure of data and year and date effects.

| Treatment group |  | |  |  |  |
| --- | --- | --- | --- | --- | --- |
| Fixed effects | Estimate | S.E. | | t-value |  |
| Intercept | 3.383 | 1.313 | | 2.576 |  |
| status (before or after the treatment) | 0.190 | 0.060 | | 3.158 |  |
| year | 0.396 | 0.332 | | 1.193 |  |
| date | -0.007 | 0.006 | | -1.350 |  |
|  |  |  | |  |  |
| Random effects |  |  | |  |  |
| *Variance parameters* |  |  | |  |  |
| site |  |  | |  | 0.252 |
| locality |  |  | |  | 0.007 |
| residual |  |  | |  | 0.038 |
| *Dimensions* |  |  | |  |  |
| # of sites. |  |  | |  | 21 |
| # of localities |  |  | |  | 5 |

Significance of fixed effects based on likelihood ratio tests: status, *P* = 0.001; year, *P* = 0.233; date, *P* = 0.177

| Control group |  | |  |  |  |
| --- | --- | --- | --- | --- | --- |
| Fixed effects | Estimate | S.E. | | t-value |  |
| Intercept | 12.516 | 9.999 | | 1.252 |  |
| status (before or after the treatment) | 0.259 | 0.132 | | 1.960 |  |
| year | -1.754 | 1.805 | | -0.972 |  |
| date | -0.046 | 0.044 | | -1.044 |  |
|  |  |  | |  |  |
| Random effects |  |  | |  |  |
| *Variance parameters* |  |  | |  |  |
| site |  |  | |  | 0.357 |
| locality |  |  | |  | < 0.001 |
| residual |  |  | |  | 0.052 |
| *Dimensions* |  |  | |  |  |
| # of sites. |  |  | |  | 6 |
| # of localities |  |  | |  | 4 |

Significance of fixed effects based on likelihood ratio tests: status, *P* = 0.050; year, *P* = 0.331; date, *P* = 0.297
