# Supplementary material for: The effect of ULV-based mosquito control on target and non-target organisms in Hungary: An experimental field study
Source: PLoS Negl Trop Dis. 2026 Oct 5;20(10):e0014140. doi: 10.1371/journal.pntd.0014140 (PMC13657401; doi:10.1371/journal.pntd.0014140)
Supplement: Supplementary file 4 [file pntd.0014140.s004.docx]

**S4 Table.** Summary statistics for the abundance (number of individuals) of different insect taxa in the malaise trap samples collected before and after insecticide spraying given separately for the treated and control sites (all sites within the same treatment group are combined) after excluding catches from Tiszafüred. *P* values are for the corresponding paired t-tests

| ***Taxa*** | ***Group*** | ***Before***  *(sum, mean ± SE)* | ***After***  *(sum, mean ± SE)* | ***%*** | ***P*** |
| --- | --- | --- | --- | --- | --- |
| *All mosquitoes* | treatment | 1147, 67.47 ± 12.82 | 681, 40.06 ± 11.44 | -40.63% | 0.008 |
|  | control | 171, 21.38 ± 5.82 | 138, 17.25 ± 4.25 | -19.3% | 0.323 |
| *Native mosq.* | treatment | 681, 40.06 ± 7.96 | 471, 27.71 ± 8.44 | -30.84% | 0.025 |
|  | control | 151, 18.88 ± 5.25 | 117, 14.62 ± 3.62 | -22.52% | 0.349 |
| *Invasive mosq.* | treatment | 466, 27.41 ± 11.35 | 210, 12.35 ± 5.99 | -54.94 % | 0.052 |
|  | control | 20, 2.5 ± 1.54 | 21, 2.62 ± 1.73 | 5 % | 0.997 |
| *Diptera* | treatment | 5583, 328.41 ± 179.75 | 2183, 128.41 ± 37.15 | -60.9 % | 0.034 |
|  | control | 1237, 206.17 ± 102.25 | 1131, 188.5 ± 126.78 | -8.57 % | 0.120 |
| *Hymenoptera* | treatment | 810, 47.65 ± 9.19 | 742, 43.65 ± 14.57 | -8.4 % | 0.210 |
|  | control | 660, 110 ± 55.42 | 536, 89.33 ± 54.02 | -18.79 % | 0.182 |
| *Hemiptera* | treatment | 520, 30.59 ± 6.44 | 331, 19.47 ± 5.39 | -36.35 % | 0.008 |
|  | control | 206, 34.33 ± 16.38 | 190, 31.67 ± 20.87 | -7.77 % | 0.103 |
| *Lepidoptera* | treatment | 270, 15.88 ± 3.84 | 120, 7.06 ± 1.84 | -55.56 % | 0.001 |
|  | control | 147, 24.5 ± 9.49 | 86, 14.33 ± 6.85 | -41.05 % | 0.255 |
| *Coleoptera* | treatment | 138, 8.12 ± 2.25 | 100, 5.88 ± 2.79 | -27.54 % | 0.002 |
|  | control | 96, 16 ± 11.7 | 110, 18.33 ± 15.95 | 14.58 % | 0.293 |
