# Supplementary material for: The effect of ULV-based mosquito control on target and non-target organisms in Hungary: An experimental field study
Source: PLoS Negl Trop Dis. 2026 Oct 5;20(10):e0014140. doi: 10.1371/journal.pntd.0014140 (PMC13657401; doi:10.1371/journal.pntd.0014140)
Supplement: Supplementary file 6 [file pntd.0014140.s008.docx]

**METHODS**

Reviewer #1:

Garamszegi et al. investigated the short-term effects of ULV deltamethrin applications in Hungary on mosquitoes and non target flying insects, employing BG Sentinel and Malaise traps, respectively. The methodology is appropriate, and the results support the conclusions. The paired before–after–control–impact (BACI) approach is robust and appropriate for assessing treatment effects under field conditions. However, for statistical clarity, the model details (random effects, transformations, sample sizes per site) should be expanded in the methods or supplementary section to ensure reproducibility.

**AUTHORS' RESPONSE: Thank you for the appreciation.**

**Random effects are (and were) presented in the supplementary material (where the results of the mixed models are given). Information on data transformation are provided in the Material and Method section (this part is now extended to the variables that are used to predict the efficiency of the ULV treatment). Sample sizes are given in Tables 1 and 2, while site-specific sample sizes could be read from the figures in terms of the number of individuals caught. Now we provide the underlying data with the submission, so one can directly assess these sample sizes from the table.**

Reviewer #2: The objectives of the study are stated clearly.

**AUTHORS' RESPONSE: No response is necessary.**

The study design is good but caome short to be appropriate because of the traps placeed in treatment sites were 3 times more than those in control sites.

**AUTHORS' RESPONSE: Using lower sample size in the control than in the treatment group is common when data collection is logistically constrained (see https://doi.org/10.1111/brv.12237). Note that in our design, each site serves as its own control (i.e., after treatment values are compared with before treatment values), so sample sizes can be considered equal in this repeated measure design. We only chose some control sites to illustrate that the detected tendencies are not transparent in those sites where no ULV treatment was applied, but we performed no statistical tests (and obtained P values) on datasets that combined control and treatment sites. Furthermore, based on the comment of Reviewer** #**1, we have performed new analyses using a reduced set of treatment sites leading to a number of treatment sites similar to that of control sites, and the main conclusions do not change: mosquito abundance still shows a considerable decline in the treatment sites, while not in the control sites. These new results have been added to the manuscript.**

Authors failed to clearly explain the sites of field work.

**AUTHORS' RESPONSE: We have added more information on the sites (i.e., that these were typical suburban residential areas). If these are still not sufficient, we would appreciate more pointers regarding the type of information the Referee wishes to read here.**

Sample size is not adequate because collection was short term, though not clearly described I believe only one day, and the timing of the trapping is also not consistence with the times Aedes mosquitoes are known to seek hosts for blood.

**AUTHORS' RESPONSE: We understand that the comment of the Referee is related to the biological focus of our study, but should not be interpreted as a methodological shortcoming. As we stated in the Introduction, our hypotheses and predictions were concerned with the immediate effect of the ULV treatment (i.e., how many individuals and species this can kill). The lethal effect of the tested chemicals lasts only few hours maximum, for which the 1-day window is appropriate. In this study, we were not interested in how the populations recover over longer time period, a question that would require longer sampling. As we do appreciate that it could be an obvious next step to investigate the mid- and long-term effects, we have mentioned this extension in the Discussion, and highlight that our results are not appropriate to make judgements on long-term effects.**

**We also showed that the reduction in the abundance of invasive Aedes mosquitoes is similar to other mosquitoes, so it suggests that the time of Aedes mosquitoes seeking hosts for blood should not be a confounder (otherwise, we had not detected comparable effects in invasive mosquitoes due to the diluting effect of daily activity whereby a given proportion of individuals would have been missed by the treatment). Note that our traps were operating for more than 24 hours, so issues raised by the activity time of Aedes mosquitoes should only have consequences for the time of ULV treatment (which was not controlled by us, as we followed the centrally organized program), if any, and not the temporal design of our trapping protocol. Our conclusion is that the ULV treatment as applied in practice in Hungary seems to be effective against invasive Aedes mosquitoes irrespective when they feed.**

Statistical analyses are okay.

**AUTHORS' RESPONSE: No response is necessary.**

No ethical concerns.

**AUTHORS' RESPONSE: No response is necessary.**

Many crucial details are missing in the methods.

**AUTHORS' RESPONSE: We have updated the Method section. We have also incorporated the suggestions of the other Referees. If these are not sufficient, we would appreciate more specific pointers about the specific details that are missing.**

Reviewer #3: No issues

**AUTHORS' RESPONSE: No response is necessary.**

**RESULTS**

Reviewer #1:

The authors report an overall reduction of ~40% in both mosquitoes and non target insects, with no difference between native and invasive mosquito species. Significant site specific variation in ULV efficacy was observed, highlighting the influence of local factors. Efficacy correlated positively with initial mosquito abundance and negatively with wind speed. In addition, the relationships for initial mosquito abundance and wind power were also significant, suggesting that wind speed may indirectly affect the efficacy of ULV by reducing mosquito abundance. Interestingly, initial mosquito abundance was significantly higher in the treated sites than in the control areas before the day of ground spraying. Could the reduced abundance in control sites affect the outcomes of the results, as the efficacy of the strategy positively correlates with mosquito abundance?.

**AUTHORS' RESPONSE: This is a plausible explanation, thank you very much for the pointer. To examine this possibility (that may undermine our conclusions), we have selected treatment sites with sample sizes that are similar to sample size of the control sites (i.e., initial mosquito abundance was lower or equal than in the control sites). In this set of samples, we could also show that the number of individuals significantly declines suggesting that reduced abundance in the control sites should not affect the results. To clarify the issue, we have added these new results to manuscript. For the Referee’s interest, we have redrawn Figure 2A by focusing only the reduced set of treatment sites (with similar abundance to that of control sites). We are not presenting this figure in the manuscript, as Figure 2A (as well as the raw datafile in the Supplement) already shows these data.**

**Please, also note that the relationship between initial mosquito abundance and the change in abundance following the treatment should represent a simple scale effect (i.e., a ~40% reduction means larger number of mosquitoes to die in places where there are more mosquitoes already, in sites where there are fewer mosquitoes the same ~40% reduction will lead to a smaller change in absolute numbers), and not that the ULV treatment is more effective at larger abundance.**

For non target organisms, small and medium sized taxa showed the strongest declines, while large bodied insects were unaffected. This pattern may reflect differences in Malaise trap capture efficiency, but could also be explained by body-size-dependent insecticide susceptibility. Larger insects typically require higher doses to achieve lethality, suggesting that ULV doses may have been insufficient. To prevent this bias, the doses are usually expressed as ng/mg body weight in topical application assays. Identifying the body size threshold at which ULV reduces insect numbers would be valuable.

Additionally, a negative trend was apparent across all major orders investigated, except for Hymenoptera. Is it associated with body size?

**AUTHORS' RESPONSE: We agree with the Reviewer’s argument, and this is why we also brought up the issue in the original manuscript. Based on our focus, design and results, we cannot distinguish between body-size-dependent insecticide susceptibility (large bodied insect need larger doses to be killed) and other body-size dependent avoidance mechanisms (larger flying insects are less likely to intercept insecticide aerosols during spraying events). In the Discussion, we consider these two explanations equally. However, the fact that we cannot resolve these issues, should not affect our main conclusion, as this is not the main focus of the study.**

**We have not measured the exact body size of each insect caught, so we are not able to estimate the body size threshold at which ULV reduces insect numbers. We have taxonomically identified more than 30,000 individuals in this study, and measuring body size for all of them would have been challenging and would not have served the main objectives of the study. What we could check is if the lack of decline in the Hymenoptera group was due to their larger body size. We can report that the (decline in the number of individuals in the large body size category)/(decline in the number of individuals in the small and medium body size category) for the Hymenoptera group is 0.022508039, while the same ratio for other insect taxonomic groups combined is 0.022503823. This is a very small difference, so we can infer that the failure to discover a negative trend in Hymenoptera was unlikely to be caused by body size effects. We decided not to include these additional results in the manuscript, as it would make an already complex story more complicated with a result that is marginal to the main hypotheses under test. However, if the Editor/Referee wishes us to do so, we remain open to make these additions.**

**Note that the results should not be mediated by differences in Malaise trap capture efficiency, because we applied the same trapping protocol before and after treatment. So, if traps have less or higher selectivity for larger insects, this effect should be the same before and after the treatment, and cannot bias the number of individuals caught along the sampling regime of our experiment.**

Reviewer #2: Yes the analysis represented match the analysis plan.

**AUTHORS' RESPONSE: No response is necessary.**

However, the results text are difficult to follow. It needs careful improvements.

**AUTHORS' RESPONSE: We have made an effort to improve the writing. We have also incorporated the suggestions of the other Relievers. If these are not sufficient, we would appreciate more specific pointers about the specific details that are missing.**

Figures need to be reduced.

**AUTHORS' RESPONSE: We have reduced the figures**

Reviewer #3: No issues

**AUTHORS' RESPONSE: No response is necessary.**

**CONCLUSIONS**

Reviewer #1:

The main limitation is the short monitoring period (24 h), which restricts conclusions. Longer term studies are needed to determine whether transient declines translate into sustained suppression or affect disease transmission.

**AUTHORS' RESPONSE: We understand that the comment of the Referee is related to the biological focus of our study, but should not be interpreted as a methodological shortcoming. As we stated in the Introduction, our hypotheses and predictions were concerned with the immediate effect of the ULV treatment (i.e., how many individuals and species this can kill). The lethal effect of the tested chemicals lasts only few hours maximum, for which the 1-day window is appropriate. In this study, our focus was not to explore how the populations recover over longer time period, a question that would require longer sampling. As we do appreciate that it could be an obvious next step to investigate the mid- and long-term effects, we have mentioned this extension in the Discussion, and highlight that our results are not appropriate to make judgements long-term effects.**

Additionally, effects on non flying insects were not assessed. Besides, only insect abundance before and after ULV was measured, while other physiological effects on target and non-target organisms, such as reproduction, were not evaluated.

**AUTHORS' RESPONSE: We also agree that this is an interesting direction, but it is beyond the scope of the study focusing on the lethal effects of the applied ULV treatment on target and non-target organisms. We now suggest in the Discussion that additional studies are needed in this direction as well.**

Overall, the limitations are clearly acknowledged in the manuscript, but further work is required to establish the mid and long term efficacy of ULV strategies and their broader ecological impact.

**AUTHORS' RESPONSE: We completely agree with this observation, and we acknowledge that further studies are needed towards this direction. Accordingly, we have started a follow up study that focuses on mid- and long-term effects.**

Reviewer #2: Conclusions are okay.

**AUTHORS' RESPONSE: No response is necessary.**

Limitations of the study are acknowledged.

**AUTHORS' RESPONSE: No response is necessary.**

Public health relevance are addressed.

**AUTHORS' RESPONSE: No response is necessary.**

Reviewer #3: Yes

**AUTHORS' RESPONSE: No response is necessary.**

**SUMMARY AND GENERAL COMMENTS**

Reviewer #3: This is a very interesting manuscript describing a well-designed pragmatic study of ULV effectiveness under real-world conditions and also impacts on non-target organisms which is far less commonly looked at. The manuscript is very well written, clear for me throughout and results are convincing. Inevitably, especially for a pragmatic study, there are liitations but the authors very clearly acknowledge these in the Discussion and I don’t believe they undermine any conclusions. A really excellent piece of work which I think could be well cited.

**AUTHORS' RESPONSE: Thank you very much for the appreciation.**

I have just a few trivial corrections and one small query below

Line 68 not incorrect but I think the more normally-used English common name is the yellow fever mosquito

Line 71 Zika is uppercase

Line 204 should read taxonomic I think

Line 391 I don’t follow the meaning of this sentence

**AUTHORS' RESPONSE: We have corrected the above issues (as suggested by another Referee, we have removed the English common names for mosquitoes).**

**REVIEWER COMMENTS IN THE ATTACHMENT FILE**

Abstract

It is important to indicate that it was deltamethrin + Chrysanthemum cinerariaefolium extract which were used for the spraying.

**AUTHORS' RESPONSE: We have added this detail.**

Introduction

- General: abstract fails to discuss if there were ever cases or outbreaks of arboviral infection within Hungary;
- and failed to show if ULV is a control method approved by WHO, and give examples of non-Hungarian sites/countries where ULV has been used before.

**AUTHORS' RESPONSE: We have added these details to the Introduction (not to the abstract). Note that the WHO does not formally approve the method, it only provides guidelines. The country-wise permissions are provided by the relevant local authorities. This is now clarified in the Method section, where it is more relevant.**

There are many studies and reviews on the effectiveness of the ULV and its non-target effect, including using BACI approach.

**AUTHORS' RESPONSE: We cite these studies in the Discussion: e.g. Boubidi et al. 2016; Corcos et al. 2020; Fonseca et al. 2013; Hart et al. 2024; Manica et al. 2017; Unlu et al. 2018. We have performed an additional literature search, and we believe that we successfully identified the most relevant literature, which are now included in the text. In case, we overlooked and important reference, we would highly appreciate more specific pointers.**

Lines 67-68: please remove the Asian tiger mosquito and the Egyptian mosquito and provide references at the end of the sentence, in line 69.

Lines 76-81: Please provide references.

Lines 83-87: references, please.

Lines 89-93: references, please.

Line 107: provide a foundational reference about BACI design/approach.

**AUTHORS' RESPONSE: We have added the appropriate references.**

Materials and Methods

General: Please cite relevant citations in the Methods. Also, many of the sentences are not easy to follow. Please paraphrase.

**AUTHORS' RESPONSE: We added more references, wherever we felt appropriate. We have also revised the writing.**

Lines 122-129: It was not clear at all what sites, where used as experiments and what sites were control sites. It is not clear at all if the authors did the spraying themselves or it’s the 3 Hungarian companies that did it. These paragraph is confusing. What has the 3 companies got to do with the study?

**AUTHORS' RESPONSE: We have clarified these issues in the text.**

If the companies have publicly available information about their organised mosquito control program in 2024 and 2024 why contact them?

**AUTHORS' RESPONSE: Due to the lack of information about the exact routes of the vehicles, as these were not available. Additionally, the spraying program can change last minute, due to weather conditions, to which we have to adjust our protocols. These fine-tuning aspects can be flexibly handled if we contact the companies and do not only rely on the publicly available information. To avoid confusion, we have removed “publicly available”, and made the whole description clearer.**

Lines 144-146: are supposed to correct for confounding factors, but it is not easy to understand what was done. Please paraphrase.

**AUTHORS' RESPONSE: We have rephased the sentence**

Line 148: 13 traps were operated in treatment sites and only four in control sites. There is a clear bias here, when analysing the data trends will skew toward the treatment sites.

**AUTHORS' RESPONSE: Using lower sample size in the control than in the treatment group is common when data collection is logistically constrained (see https://doi.org/10.1111/brv.12237). Note that in our design each site serves as its own control (i.e., after treatment values are compared with before treatment values), so sample sizes can be considered equal in this repeated measure design. We only chose some control sites to illustrate that the detected tendencies are not transparent in those sites where no ULV treatment was applied, but we performed no statistical tests (and obtained P values) on datasets that combined control and treatment sites. Furthermore, based on the comment of Reviewer** #**1, we have performed new analyses using a reduced set of treatment sites leading to a number of treatment sites similar to that of control sites, and the main conclusions do not change: mosquito abundance still shows a considerable decline in the treatment sites, while not in the control sites. These new results have been added to the manuscript.**

Line 176-178: ULV application between 10:00pm and 02:00 am, in what months? And these times of ULV application are not optimal for the known host seeking behaviour of Aedes mosquitoes.

**AUTHORS' RESPONSE: We have added the details on the timing.**

**We showed that the reduction in the abundance of invasive Aedes mosquitoes is similar to other mosquitoes, so it suggests that the time of Aedes mosquitoes seeking hosts for blood is not a confounder. Note that our traps were operating for more than 24 hours, so issues raised by the activity time of Aedes mosquitoes should have consequences for the time of ULV treatment (which was not controlled by us, as we followed the centrally organized program), if any, and not the temporal design of our trapping protocol. Our conclusion is that the ULV treatment that is applied in practice in Hungary seems to be effective against invasive Aedes mosquitoes regardless of when they feed.**

Line 188: we installed, not we aimed to install? Please check line 196 as well.

**AUTHORS' RESPONSE: We have corrected the sentence**

Lines 199-203: Samplings should have been done in 24 and 48 hour, not only one time point.

**AUTHORS' RESPONSE: Indeed, we have used this sampling window (i.e., the traps were operating 24 hours before collection and not one time point), as shown in Figure 1. We may have misunderstood the Reviewer's comment and would therefore appreciate further clarification.**

Lines 243-246: temperature, humidity and windspeed during trapping should provide more information than daily averages.

**AUTHORS' RESPONSE: These are not daily averages, these are the accuracies of the measurements, which are presented according to the scientific standards. We may have misunderstood the Reviewer's comment and would therefore appreciate further clarification.**

Lines 249-287: This is too long. Please reduce it by half.

**AUTHORS' RESPONSE: We are confused. Above there were several unspecified comments that suggested that more details are needed in the Method section. Here, without any specific detail, it is suggested that this methodological section should be reduced. This is hard to follow that Referees conflicting comments when no particular pointers are provided (see also the next comment). We rely on the Editor's suggestion in such situation.**

Results

General: Results did not explain clearly how the results were grounded on BACI design.

**AUTHORS' RESPONSE: We have described in detail the BACI design in the Method section), and then, present the results that are coming out from that design. So, the results were entirely grounded on the BACI design as explained in the Method. We feel that the current and the above comment of the Referee are in conflict with each other: here further explanations are requested, while above we are asked to make reduction in the corresponding method section.**

Line 292-293. What is the basic of results with ratio? Is it because of collections per hour, per day, or average for all the sites? This is not clear. Authors should also refer to mosquitoes as mosquitoes not individuals.

**AUTHORS' RESPONSE: We have clarified how we calculated these ratios (proportions of the total number of individuals captured in the traps before and after treatment during the entire 24-hour sampling period). We have also clarified it for Table 2.**

Figure 2; What mosquito species are endemic and which ones are invasive?

**AUTHORS' RESPONSE: We have provided this information in the figure legend. We also specify it on Table 1**

Line 294: In the abstract authors said 45% decline and here it is ~47%?

**AUTHORS' RESPONSE: We have corrected it in the abstract.**

Line 305: When analysed separately the results from native and invasive species were in similar or were concordant. Please rephrase. We found parallel results is not good enough.

**AUTHORS' RESPONSE: We have rephased the sentence.**

Line 310: The number of invasive mosquito species was also…. Please remove individual.

**AUTHORS' RESPONSE: The Referee may have misunderstood what we meant to say here. We have rephrased the sentence to avoid confusion.**

Lines 317-319: Sentence not clear. Please endeavour to rephrase this. What are signatures?

**AUTHORS' RESPONSE: We have rephased the sentence.**

Line 320: at particular treatment sites? What sites? You can say x number of sites or name them please.

**AUTHORS' RESPONSE: We mean all of them. We have clarified it.**

Line 321-329: Figure 3B not cited.

**AUTHORS' RESPONSE: Now we cite it (using the new figure numbers, as suggested below).**

Line 333: There are so many figures in this manuscript. Please make the lone figure 4 into figure 3C so that you have less figure numbers. Figure 5 and 6 and 7 can be changed into figures 4 and 5 and 6 respectively, etc.

**AUTHORS' RESPONSE: Figure 4 addresses a conceptually different aspect of the study than Figure 3, and we therefore felt that combining these two figures would reduce their clarity. Specifically, Figure 3 presents changes in mosquito abundance, whereas Figure 4 focuses on the effects on non-target organisms. To accommodate the Reviewer's suggestion as much as possible, we instead reorganized the figures by combining Figures 2 and 3, and Figures 4, 5 and 7.**

Discussion

Line 423: Wind speed not wing speed?

**AUTHORS' RESPONSE: Sorry for the typo, we have corrected it.**
