# Supplementary material for: The effect of ULV-based mosquito control on target and non-target organisms in Hungary: An experimental field study
Source: PLoS Negl Trop Dis. 2026 Oct 5;20(10):e0014140. doi: 10.1371/journal.pntd.0014140 (PMC13657401; doi:10.1371/journal.pntd.0014140)
Supplement: Supplementary file 7 [file pntd.0014140.s006.docx]

**Review PNTD-D-26-00481: The effect of ULV-based mosquito control on target and non-target organisms in Hungary: an experimental field study**

**Abstract**

It is important to indicate that it was deltamethrin + Chrysanthemum cinerariaefolium extract which were used for the spraying**.**

**Introduction**

General: abstract fails to

(i) discuss if there were ever cases or outbreaks of arboviral infection within Hungary; and (
(ii) failed to show if ULV is a control method approved by WHO, and give examples of non-Hungarian sites/countries where ULV has been used before.

There are many studies and reviews on the effectiveness of the ULV and its non-target effect, including using BACI approach.

Lines 67-68: please remove the Asian tiger mosquito and the Egyptian mosquito and provide references at the end of the sentence, in line 69.

Lines 76-81: Please provide references.

Lines 83-87: references, please.

Lines 89-93: references, please.

Line 107: provide a foundational reference about BACI design/approach.

**Materials and Methods**

General: Please cite relevant citations in the Methods. Also, many of the sentences are not easy to follow. Please paraphrase.

Lines 122-129: It was not clear at all what sites where used as experiments and what sites were control sites. It is not clear at all if the authors did the spraying themselves or it’s the 3 Hungarian companies that did it. These paragraph is confusing. What has the 3 companies got to do with the study? If the companies have publicly available information about their organised mosquito control program in 2024 and 2024 why contact them?

Lines 144-146: are supposed to correct for confounding factors, but it is not easy to understand what was done. Please paraphrase.

Line 148: 13 traps were operated in treatment sites and only four in control sites. There is a clear bias here, when analysing the data trends will skew toward the treatment sites.

Line 176-178: ULV application between 10:00pm and 02:00 am, in what months? And these times of ULV application are not optimal for the known host seeking behaviour of Aedes mosquitoes.

Line 188: we installed, not we aimed to install? Please check line 196 as well.

Lines 199-203: Samplings should have been done in 24 and 48 hour, not only one time point.

Lines 243-246: temperature, humidity and windspeed during trapping should provide more information than daily averages.

Lines 249-287: This is too long. Please reduce it by half.

**Results**

General: Results did not explain clearly how the results were grounded on BACI design.

Line 292-293. What is the basic of results with ratio? Is it because of collections per hour, per day, or average for all the sites? This is not clear. Authors should also refer to mosquitoes as mosquitoes not individuals.

Figure 2; What mosquito species are endemic and which ones are invasive?

Line 294: In the abstract authors said 45% decline and here it is ~47%?

Line 305: When analysed separately the results from native and invasive species were in similar or were concordant. Please rephrase. We found parallel results is not good enough.

Line 310: The number of invasive mosquito species was also…. Please remove individual.

Lines 317-319: Sentence not clear. Please endeavour to rephrase this. What are signatures?

Line 320: at particular treatment sites? What sites? You can say x number of sites or name them please.

Line 321-329: Figure 3B not cited.

Line 333: There are so many figures in this manuscript. Please make the lone figure 4 into figure 3C so that you have less figure numbers. Figure 5 and 6 and 7 can be changed into figures 4 and 5 and 6 respectively, etc.

**Discussion**

Line 423: Wind speed not wing speed?
