# Supplementary material for: The effect of ULV-based mosquito control on target and non-target organisms in Hungary: An experimental field study
Source: PLoS Negl Trop Dis. 2026 Oct 5;20(10):e0014140. doi: 10.1371/journal.pntd.0014140 (PMC13657401; doi:10.1371/journal.pntd.0014140)
Supplement: Peer review history

# Peer review: Decision Letter 0

**DOI:** 10.1371/journal.pntd.0014140.r001

**Authors:** Álvaro Acosta-Serrano

16 Jul 2026

PNTD-D-26-00481The effect of ULV-based mosquito control on target and non-target organisms in Hungary: an experimental field studyPLOS Neglected Tropical Diseases Dear Dr. Garamszegi,Thank you for submitting your manuscript to PLOS Neglected Tropical Diseases. After careful consideration, we feel that it has merit but does not fully meet PLOS Neglected Tropical Diseases's publication criteria as it currently stands. Therefore, we invite you to submit a revised version of the manuscript that addresses the points raised during the review process.Please submit your revised manuscript by Sep 14 2026 11:59PM. If you will need more time than this to complete your revisions, please reply to this message or contact the journal office at plosntds@plos.org. When you're ready to submit your revision, log on to https://www.editorialmanager.com/pntd/ and select the 'Submissions Needing Revision' folder to locate your manuscript file.Please include the following items when submitting your revised manuscript:* A letter that responds to each point raised by the editor and reviewer(s). You should upload this letter as a separate file labeled 'Response to Reviewers'. This file does not need to include responses to any formatting updates and technical items listed in the 'Journal Requirements' section below.* A marked-up copy of your manuscript that highlights changes made to the original version. You should upload this as a separate file labeled 'Revised Manuscript with Track Changes'.* An unmarked version of your revised paper without tracked changes. You should upload this as a separate file labeled 'Manuscript'.If you would like to make changes to your financial disclosure, competing interests statement, or data availability statement, please make these updates within the submission form at the time of resubmission. Guidelines for resubmitting your figure files are available below the reviewer comments at the end of this letter.

As the corresponding author, your ORCID iD is verified in the submission system and will appear in the published article. PLOS supports the use of ORCID, and we encourage all coauthors to register for an ORCID iD and use it as well. Please encourage your coauthors to verify their ORCID iD within the submission system before final acceptance, as unverified ORCID iDs will not appear in the published article. Only the individual author can complete the verification step; PLOS staff cannot verify ORCID iDs on behalf of authors.

We look forward to receiving your revised manuscript.

Kind regards,Álvaro Acosta-SerranoSection EditorPLOS Neglected Tropical Diseases

Shaden Kamhawi

co-Editor-in-Chief

PLOS Neglected Tropical Diseases

orcid.org/0000-0003-4304-636XX

Paul Brindley

co-Editor-in-Chief

PLOS Neglected Tropical Diseases

orcid.org/0000-0003-1765-0002

**Additional Editor Comments (if provided):**  **Journal Requirements:**

If the reviewer comments include a recommendation to cite specific previously published works, please review and evaluate these publications to determine whether they are relevant and should be cited. There is no requirement to cite these works unless the editor has indicated otherwise.

1) Please ensure that the CRediT author contributions listed for every co-author are completed accurately and in full.

At this stage, the following Authors/Authors require contributions: Laszlo Garamszegi, Gergely Nagy, Ágnes Klein, Tamara Szentiványi, Zsóka Vásárhelyi, Gábor Markó, Sándor Zsebők, and Zoltán Soltész. Please ensure that the full contributions of each author are acknowledged in the "Add/Edit/Remove Authors" section of our submission form.

The list of CRediT author contributions may be found here: https://journals.plos.org/plosntds/s/authorship#loc-author-contributions

2) Please provide an Author Summary. This should appear in your manuscript between the Abstract (if applicable) and the Introduction, and should be 150-200 words long. The aim should be to make your findings accessible to a wide audience that includes both scientists and non-scientists. Sample summaries can be found on our website under Submission Guidelines:

https://journals.plos.org/plosntds/s/submission-guidelines#loc-parts-of-a-submission

3) Please upload all main figures as separate Figure files in .tif or .eps format. For more information about how to convert and format your figure files please see our guidelines:

https://journals.plos.org/plosntds/s/figures

4) We notice that your supplementary Tables are included in the manuscript file. Please remove them and upload them with the file type 'Supporting Information'. Please ensure that each Supporting Information file has a legend listed in the manuscript after the references list.

5) In the online submission form, you indicated that Data will be made available in Dyrad upon acceptance. All PLOS journals now require all data underlying the findings described in their manuscript to be freely available to other researchers, either

1. In a public repository

2. Within the manuscript itself

3. Uploaded as supplementary information.

This policy applies to all data except where public deposition would breach compliance with the protocol approved by your research ethics board. If your data cannot be made publicly available for ethical or legal reasons (e.g., public availability would compromise patient privacy), please explain your reasons by return email and your exemption request will be escalated to the editor for approval. Your exemption request will be handled independently and will not hold up the peer review process, but will need to be resolved should your manuscript be accepted for publication. One of the Editorial team will then be in touch if there are any issues.

6) Please amend your detailed Financial Disclosure statement. This is published with the article. It must therefore be completed in full sentences and contain the exact wording you wish to be published.

1) State what role the funders took in the study. If the funders had no role in your study, please state: "The funders had no role in study design, data collection and analysis, decision to publish, or preparation of the manuscript."

2) If any authors received a salary from any of your funders, please state which authors and which funders..

If you did not receive any funding for this study, please simply state: u201cThe authors received no specific funding for this work.u201d

7) Your current Financial Disclosure states, "The study was supported by funds from Hungary's National Research, Development and Innovation Office (K135841, RRF-2.3.1-21-2022-00006, ADVANCED 152427) and by the AXA Research Fund. G.M. was supported by the Research Excellence Programme of the Hungarian University of Agriculture and Life Sciences (KKP2024-MG, KKP2026-MG). Z.S was supported by the National Research, Development and Innovation Office (FK-147466)." However, your funding information on the submission form indicates "order of grants". Please indicate by return email the full and correct funding information for your study and confirm the order in which funding contributions should appear. Please be sure to indicate whether the funders played any role in the study design, data collection and analysis, decision to publish, or preparation of the manuscript.

8) We have amended your Competing Interest statement to comply with journal style. We kindly ask that you double check the statement and let us know if anything is incorrect.

**Reviewers' comments:**  Reviewer's Responses to Questions

**Key Review Criteria Required for Acceptance?**

As you describe the new analyses required for acceptance, please consider the following:

**Methods**

-Are the objectives of the study clearly articulated with a clear testable hypothesis stated?

-Is the study design appropriate to address the stated objectives?

-Is the population clearly described and appropriate for the hypothesis being tested?

-Is the sample size sufficient to ensure adequate power to address the hypothesis being tested?

-Were correct statistical analysis used to support conclusions?

-Are there concerns about ethical or regulatory requirements being met?

Reviewer #1: Garamszegi et al. investigated the short-term effects of ULV deltamethrin applications in Hungary on mosquitoes and non target flying insects, employing BG Sentinel and Malaise traps, respectively. The methodology is appropriate, and the results support the conclusions.

The paired before–after–control–impact (BACI) approach is robust and appropriate for assessing treatment effects under field conditions. However, for statistical clarity, the model details (random effects, transformations, sample sizes per site) should be expanded in the methods or supplementary section to ensure reproducibility.

Reviewer #2: The objectives of the study are stated clearly.

The study design is good but caome short to be appropriate because of the traps placeed in treatment sites were 3 times more than those in control sites.

Authors failed to clearly explain the sites of field work.

Sample size is not adequate because collection was short term, though not clearly described I believe only one day, and the timing of the trapping is also not consistence with the times Aedes mosquitoes are known to seek hosts for blood.

Statistical analyses are okay.

No ethical concerns.

Many crucial details are missing in the methods.

Reviewer #3: No issues

**********

**Results**

-Does the analysis presented match the analysis plan?

-Are the results clearly and completely presented?

-Are the figures (Tables, Images) of sufficient quality for clarity?

Reviewer #1: The authors report an overall reduction of ~40% in both mosquitoes and non target insects, with no difference between native and invasive mosquito species. Significant site specific variation in ULV efficacy was observed, highlighting the influence of local factors. Efficacy correlated positively with initial mosquito abundance and negatively with wind speed. In addition, the relationships for initial mosquito abundance and wind power were also significant, suggesting that wind speed may indirectly affect the efficacy of ULV by reducing mosquito abundance. Interestingly, initial mosquito abundance was significantly higher in the treated sites than in the control areas before the day of ground spraying. Could the reduced abundance in control sites affect the outcomes of the results, as the efficacy of the strategy positively correlates with mosquito abundance?.

For non target organisms, small and medium sized taxa showed the strongest declines, while large bodied insects were unaffected. This pattern may reflect differences in Malaise trap capture efficiency, but could also be explained by body-size-dependent insecticide susceptibility. Larger insects typically require higher doses to achieve lethality, suggesting that ULV doses may have been insufficient. To prevent this bias, the doses are usually expressed as ng/mg body weight in topical application assays. Identifying the body size threshold at which ULV reduces insect numbers would be valuable.

Additionally, a negative trend was apparent across all major orders investigated, except for Hymenoptera. Is it associated with body size?

Reviewer #2: Yes the analysis represented match the analysis plan.

However, the results text are difficult to follow. It needs careful improvements.

Figures need to be reduced.

Reviewer #3: No issues

**********

**Conclusions**

-Are the conclusions supported by the data presented?

-Are the limitations of analysis clearly described?

-Do the authors discuss how these data can be helpful to advance our understanding of the topic under study?

-Is public health relevance addressed?

Reviewer #1: The main limitation is the short monitoring period (24 h), which restricts conclusions. Longer term studies are needed to determine whether transient declines translate into sustained suppression or affect disease transmission. Additionally, effects on non flying insects were not assessed. Besides, only insect abundance before and after ULV was measured, while other physiological effects on target and non-target organisms, such as reproduction, were not evaluated.

Overall, the limitations are clearly acknowledged in the manuscript, but further work is required to establish the mid and long term efficacy of ULV strategies and their broader ecological impact.

Reviewer #2: Conclusions are okay.

Limitations of the study are acknowledged.

Public health relevance are addressed.

Reviewer #3: Yes

**********

**Editorial and Data Presentation Modifications?**

Use this section for editorial suggestions as well as relatively minor modifications of existing data that would enhance clarity. If the only modifications needed are minor and/or editorial, you may wish to recommend “Minor Revision” or “Accept”.

Reviewer #1: (No Response)

Reviewer #2: Major revision.

Reviewer #3: (No Response)

**********

**Summary and General Comments**

Use this section to provide overall comments, discuss strengths/weaknesses of the study, novelty, significance, general execution and scholarship. You may also include additional comments for the author, including concerns about dual publication, research ethics, or publication ethics. If requesting major revision, please articulate the new experiments that are needed.

Reviewer #1: (No Response)

Reviewer #2: (No Response)

Reviewer #3: This is a very interesting manuscript describing a well-designed pragmatic study of ULV effectiveness under real-world conditions and also impacts on non-target organisms which is far less commonly looked at. The manuscript is very well written, clear for me throughout and results are convincing. Inevitably, especially for a pragmatic study, there are liitations but the authors very clearly acknowledge these in the Discussion and I don’t believe they undermine any conclusions. A really excellent piece of work which I think could be well cited. I have just a few trivial corrections and one small query below

Line 68 not incorrect but I think the more normally-used English common name is the yellow fever mosquito

Line 71 Zika is uppercase

Line 204 should read taxonomic I think

Line 391 I don’t follow the meaning of this sentence

**********

PLOS authors have the option to publish the peer review history of their article (what does this mean?). If published, this will include your full peer review and any attached files.

If you choose “no”, your identity will remain anonymous but your review may still be made public.

**Do you want your identity to be public for this peer review?**  For information about this choice, including consent withdrawal, please see our Privacy Policy.

Reviewer #1: **Yes:**  Marcos Sterkel

Reviewer #2: No

Reviewer #3: No

[NOTE: If reviewer comments were submitted as an attachment file, they will be attached to this email and accessible via the submission site. Please log into your account, locate the manuscript record, and check for the action link "View Attachments". If this link does not appear, there are no attachment files.] **Figure resubmission:**   While revising your submission, we strongly recommend that you use PLOS’s NAAS tool (https://ngplosjournals.pagemajik.ai/artanalysis) to test your figure files. NAAS can convert your figure files to the TIFF file type and meet basic requirements (such as print size, resolution), or provide you with a report on issues that do not meet our requirements and that NAAS cannot fix.

After uploading your figures to PLOS’s NAAS tool - https://ngplosjournals.pagemajik.ai/artanalysis, NAAS will process the files provided and display the results in the "Uploaded Files" section of the page as the processing is complete. If the uploaded figures meet our requirements (or NAAS is able to fix the files to meet our requirements), the figure will be marked as "fixed" above. If NAAS is unable to fix the files, a red "failed" label will appear above. When NAAS has confirmed that the figure files meet our requirements, please download the file via the download option, and include these NAAS processed figure files when submitting your revised manuscript. **Reproducibility:**  To enhance the reproducibility of your results, we recommend that authors of applicable studies deposit laboratory protocols in protocols.io, where a protocol can be assigned its own identifier (DOI) such that it can be cited independently in the future. Additionally, PLOS ONE offers an option to publish peer-reviewed clinical study protocols. Read more information on sharing protocols at https://plos.org/protocols?utm_medium=editorial-email&utm_source=authorletters&utm_campaign=protocols

Attachment Submitted filename: Review PNTD-D-26-00481.docx

# Author comment: Author response to Decision Letter 1

**DOI:** 10.1371/journal.pntd.0014140.r002

5 Aug 2026

Attachment Submitted filename: responses_to_referees.docx

# Peer review: Decision Letter 1

**DOI:** 10.1371/journal.pntd.0014140.r003

**Authors:** Álvaro Acosta-Serrano

21 Sep 2026

Dear Dr. Garamszegi,

We are pleased to inform you that your manuscript 'The effect of ULV-based mosquito control on target and non-target organisms in Hungary: an experimental field study' has been provisionally accepted for publication in PLOS Neglected Tropical Diseases.

Before your manuscript can be formally accepted you will need to complete some formatting changes, which you will receive in a follow up email. A member of our team will be in touch with a set of requests.

Please note that your manuscript will not be scheduled for publication until you have made the required changes, so a swift response is appreciated.

IMPORTANT: The editorial review process is now complete. PLOS will only permit corrections to spelling, formatting or significant scientific errors from this point onwards. Requests for major changes, or any which affect the scientific understanding of your work, will cause delays to the publication date of your manuscript.

Should you, your institution's press office or the journal office choose to press release your paper, you will automatically be opted out of early publication. We ask that you notify us now if you or your institution is planning to press release the article. All press must be co-ordinated with PLOS.

Thank you again for supporting Open Access publishing; we are looking forward to publishing your work in PLOS Neglected Tropical Diseases.

Best regards,

Álvaro Acosta-Serrano

Section Editor

PLOS Neglected Tropical Diseases

Álvaro Acosta-Serrano

Section Editor

PLOS Neglected Tropical Diseases

Shaden Kamhawi

co-Editor-in-Chief

PLOS Neglected Tropical Diseases

orcid.org/0000-0003-4304-636XX

Paul Brindley

co-Editor-in-Chief

PLOS Neglected Tropical Diseases

orcid.org/0000-0003-1765-0002

***********************************************************

Reviewer's Responses to Questions

**Key Review Criteria Required for Acceptance?**

As you describe the new analyses required for acceptance, please consider the following:

**Methods**

-Are the objectives of the study clearly articulated with a clear testable hypothesis stated?

-Is the study design appropriate to address the stated objectives?

-Is the population clearly described and appropriate for the hypothesis being tested?

-Is the sample size sufficient to ensure adequate power to address the hypothesis being tested?

-Were correct statistical analysis used to support conclusions?

-Are there concerns about ethical or regulatory requirements being met?

Reviewer #1: All previously raised issues and concerns have been addressed in the current manuscript version.

Reviewer #2: I am not able to confirm this.

**********

**Results**

-Does the analysis presented match the analysis plan?

-Are the results clearly and completely presented?

-Are the figures (Tables, Images) of sufficient quality for clarity?

Reviewer #1: All previously raised issues and concerns have been addressed in the current manuscript version.

Reviewer #2: I am not able to verify this.

**********

**Conclusions**

-Are the conclusions supported by the data presented?

-Are the limitations of analysis clearly described?

-Do the authors discuss how these data can be helpful to advance our understanding of the topic under study?

-Is public health relevance addressed?

Reviewer #1: All previously raised issues and concerns have been addressed in the current manuscript version.

Reviewer #2: I am not able to verify this.

**********

**Editorial and Data Presentation Modifications?**

Use this section for editorial suggestions as well as relatively minor modifications of existing data that would enhance clarity. If the only modifications needed are minor and/or editorial, you may wish to recommend “Minor Revision” or “Accept”.

Reviewer #1: All previously raised issues and concerns have been addressed in the current manuscript version.

Reviewer #2: I am not able to verify this.

**********

**Summary and General Comments**

Use this section to provide overall comments, discuss strengths/weaknesses of the study, novelty, significance, general execution and scholarship. You may also include additional comments for the author, including concerns about dual publication, research ethics, or publication ethics. If requesting major revision, please articulate the new experiments that are needed.

Reviewer #1: All previously raised issues and concerns have been addressed in the current manuscript version.

Reviewer #2: I am not able to assess this resubmission because corrections are shared not as tracked changes and responses to my queries were without line numbering citations.

**********

PLOS authors have the option to publish the peer review history of their article (what does this mean?). If published, this will include your full peer review and any attached files.

If you choose “no”, your identity will remain anonymous but your review may still be made public.

**Do you want your identity to be public for this peer review?**  For information about this choice, including consent withdrawal, please see our Privacy Policy.

Reviewer #1: **Yes:**  Marcos Sterkel

Reviewer #2: No

# Editor report: Acceptance letter

**DOI:** 10.1371/journal.pntd.0014140.r004

**Authors:** Álvaro Acosta-Serrano

Dear Dr. Garamszegi,

We are delighted to inform you that your manuscript, "The effect of ULV-based mosquito control on target and non-target organisms in Hungary: an experimental field study," has been formally accepted for publication in PLOS Neglected Tropical Diseases.

We have now passed your article onto the PLOS Production Department who will complete the rest of the publication process. All authors will receive a confirmation email upon publication.

The corresponding author will soon be receiving a typeset proof for review, to ensure errors have not been introduced during production. Please review the PDF proof of your manuscript carefully, as this is the last chance to correct any scientific or type-setting errors. Please note that major changes, or those which affect the scientific understanding of the work, will likely cause delays to the publication date of your manuscript. Note: Proofs for Front Matter articles (Editorial, Viewpoint, Symposium, Review, etc...) are generated on a different schedule and may not be made available as quickly.

Soon after your final files are uploaded, the early version of your manuscript will be published online unless you opted out of this process. The date of the early version will be your article's publication date. The final article will be published to the same URL, and all versions of the paper will be accessible to readers.

For Research Articles, you will receive an invoice from PLOS for your publication fee after your manuscript has reached the completed accept phase. If you receive an email requesting payment before acceptance or for any other service, this may be a phishing scheme. Learn how to identify phishing emails and protect your accounts at https://explore.plos.org/phishing.

Thank you again for supporting open-access publishing; we are looking forward to publishing your work in PLOS Neglected Tropical Diseases.

Best regards,

Shaden Kamhawi

co-Editor-in-Chief

PLOS Neglected Tropical Diseases

Paul Brindley

co-Editor-in-Chief

PLOS Neglected Tropical Diseases
